# Preprocess dependence of optical properties of ensembles and single siphonaxanthin-containing major antenna from the marine green alga *Codium fragile*

**DOI:** 10.1038/s41598-022-11572-3

**Published:** 2022-05-19

**Authors:** Tatas Hardo Panintingjati Brotosudarmo, Bernd Wittmann, Soichiro Seki, Ritsuko Fujii, Jürgen Köhler

**Affiliations:** 1 Spectroscopy of Soft Matter, University of Bayreuth, 95440 Bayreuth, Germany; 2 Department of Food Technology, Universitas Ciputra, Citraland CBD Boulevard, Surabaya, 60219 Indonesia; 3 Graduate School of Science, Osaka City University, 3-3-138 Sugimoto, Sumiyoshi-ku, Osaka, 558-8585 Japan; 4 Research Center for Artificial Photosynthesis, Osaka Metropolitan University, 3-3-138 Sugimoto, Sumiyoshi-ku, Osaka, 558-8585 Japan; 5 Bavarian Polymer Institute, University of Bayreuth, 95440 Bayreuth, Germany; 6 Bayreuther Institut für Makromolekülforschung (BIMF), 95440 Bayreuth, Germany

**Keywords:** Biophysics, Optics and photonics

## Abstract

The siphonaxanthin-siphonein-Chl-*a/b*-protein (SCP) is the light-harvesting complex of the marine alga *Codium fragile*. Its structure resembles that of the major light-harvesting complexes of higher plants, LHC II, yet it features a reversed Chl *a*:Chl *b* ratio and it accommodates other variants of carotenoids. We have recorded the fluorescence emission spectra and fluorescence lifetimes from ensembles and single SCP complexes for three different scenarios of handling the samples. While the data obtained from ensembles of SCP complexes yield equivalent results, those obtained from single SCP complexes featured significant differences as a function of the sample history. We ascribe this discrepancy to the different excitation intensities that have been used for ensemble and single complex spectroscopy, and conclude that the SCP complexes undergo an aging process during storage. This process is manifested as a lowering of energetic barriers within the protein, enabling thermal activation of conformational changes at room temperature. This in turn leads to the preferential population of a red-shifted state that features a significant decrease of the fluorescence lifetime.

## Introduction

**Figure 1:**
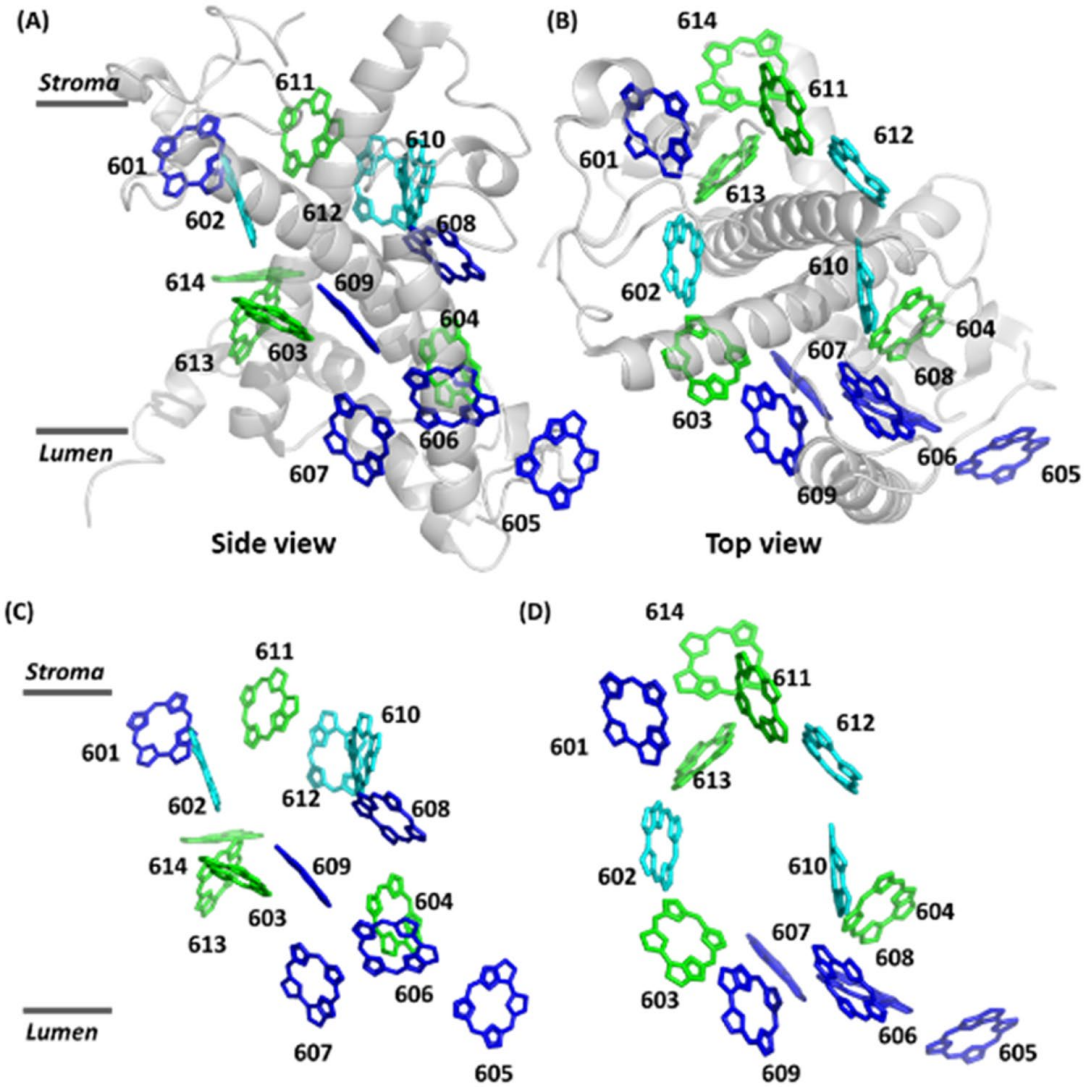
High-resolution structure of a monomer of LHC II from spinach [PDB:1RWT]. (**A, C**) Side view of the arrangement of the Chl molecules with (**A**) and without (**C**) the protein backbone. (**B, D**) Top view of the arrangement of the Chl molecules with (**B**) and without (**D**) the protein backbone. Chl a molecules are shown in green, Chl b molecules are show in blue. For clarity the carotenoids have been omitted. For SCP the Chl a molecules in position 602, and either those in positions 610 or 612 are exchanged to Chl b as indicated by the cyan colour. The Chl molecules are numbered according to the scheme given in^9^ (1RWT). The figure has been produced using PyMOL ver. 2.5.0.

Photosynthesis is exploited by plants, bacteria, and algae to convert solar energy into biochemical energy. This is achieved by the absorption of sunlight in specialised light-harvesting complexes (LHCs), and subsequent ultrafast and efficient transport of the excitation energy to a photochemical reaction centre (RC) that acts as transducer driving a transmembrane charge separation^1^. Since the prerequisites that have to be fulfilled for electron transfer are quite demanding, the structure of the RCs is mainly conserved across the various organisms that perform photosynthesis. In contrast, the rules for energy transfer are rather tolerant resulting in a rich variety for the structures of the light harvesting systems allowing for perfect adaption to the special conditions that apply to the habitat of the organism^2^. In contrast to the LHCs from higher plants the antennae systems from marine algae have only recently attracted more attention^3–7^. *Codium fragile* is a marine alga that occurs in open coasts and tidal pools, but it can also be found under water in a depth of up to about 20 m. Its LHC is the siphonaxanthin-siphonein-Chl-*a/b*-protein (SCP) that is optimized for using blue-green light, which is the dominating spectral range under water. The structure of this complex is still elusive but given the high sequence identity with the major LHC II complex from green plants^7^ the idea is that the structure of SCP resembles that of LHC II, which is known with atomic resolution from x-ray crystallography^8,9^. LHC II is a heterotrimer and each monomer binds 8 Chl *a*, 6 Chl *b*, and 4 molecules of carotenoid (Car), namely two non-equivalent Luteins (Lut), one 9'-cis neoxanthin (Neo), and one Violaxanthin (Vio) or Zeaxanthin (Zea)^9^. For SCP it is known that is forms as well a trimeric complex that accommodates Chl *a*, Chl *b*, and that the two Lut molecules are replaced by siphonaxanthin (Sx) and its esterified variant siphonein (Sn), respectively, and 9'-cis neoxanthin (Neo). Recently, a ratio of (Sx + Sn):Neo:Chl *a*:Chl *b* = 2.5:1:6:8 was obtained by HPLC analysis for the pigment structure from resonance Raman spectroscopy^4^. A similar Chl *a*:Chl *b* ratio was determined earlier also for the LHCs of PS II from the siphonous green algae *Bryopsis maxima* and *Bryopsis corticulans*^6,7^. In summary, in SCP the two molecules of Lut in LHC II are replaced by Sx/Sn, Vio is not present, and the Chl *a* to Chl *b* ratio is reversed with respect to that in LHC II. Both the exchange of the carotenoids and the larger amount of Chl *b* molecules enhance the light-harvesting efficiency of the complex in the blue-green spectral region around 450 nm, which reflects the adaption of the species to the natural habitat. Since the ligands in the Chl binding pockets are the same in LHC II and SCP^7^ the reversed Chl *a* to Chl *b* ratio hints for some structural flexibility of the SCP complex at these positions. Based on recent cryoEM data^10^ the Chl *a* to Chl *b* exchange sites are assigned to the position 602, and either to positions 610 or 612, see Fig. 1, according to the numbering scheme used in^9^.

Usually, the details in the optical spectra from a macroscopic ensemble of proteins are washed out due to ensemble averaging. In contrast, studying the complexes individually provides information about the distributions of parameters rather than only about their moments, which allows to identify subpopulations that would be obscured otherwise. In the past, single-molecule spectroscopy has been applied extensively to the antennae complexes of purple bacteria^11–20^ and to the light-harvesting systems associated with plant photosystems I and II^21–29^ for obtaining information about the energetics and dynamics both at cryogenic temperatures as well as under ambient conditions, recently excellently reviewed in^30^. At low temperatures thermal motions of the nuclei are frozen out and the electronic transition energies that serve as input for structure-based modelling can be detected with enhanced spectral resolution. On the other hand, experiments under ambient conditions are closer to the natural environment and provide valuable information about the conformational dynamics of the pigment-protein complexes. In particular, a single protein that undergoes conformational transitions is at any time in a distinct, well-defined state in contrast to an ensemble of proteins due to the lack of synchronization^31^. To the best of our knowledge results from optical spectroscopy on single SCP complexes have not been published to date.

Here we report about spectroscopy on ensembles and single trimeric SCP complexes at room temperature for three different protocols for handling the samples. The first protocol (protocol 1) refers to fresh samples prepared on ice under ambient conditions, the second protocol (protocol 2) refers to SCP complexes from the same batch that have been stored for 7 months at − 80 °C in the dark, and that are prepared under the same conditions as before. The last protocol (protocol 3) refers to experiments where the samples from the 7 months old SCP complexes have been prepared in a cold room at 5 °C. While the absorption and emission spectra from ensembles of SCP complexes in solution featured a high degree of similarity for the three protocols, the single complex approach revealed strong differences in the spectral details as a function of the sample preparation history.

## Results

**Figure 2:**
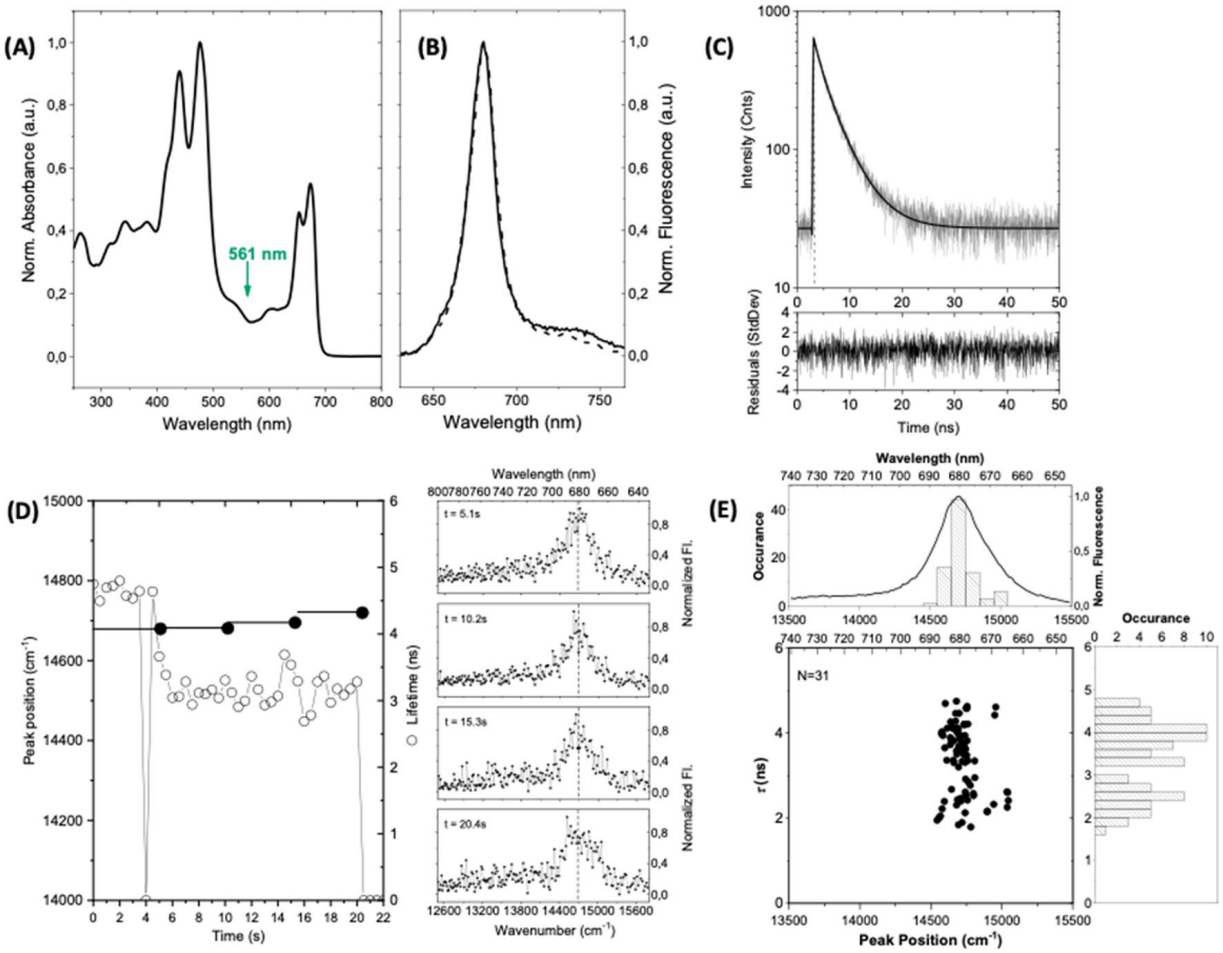
Spectroscopy on ensembles and single SCP complexes. The samples for spectroscopy have been prepared under ambient conditions (protocol 1). (**A**) Room temperature absorption spectrum of an ensemble of SCP complexes dissolved in bulk buffer solution at a concentration of 1.45 × 10^–7^ M. (**B**) Peak normalised emission spectra from the bulk buffer solution for an excitation intensity of 30 μW/cm^2^ (full line) and from an ensemble of SCP complexes embedded in a thin film of PVA (dashed line) for an excitation intensity of 525 W/cm^2^. The excitation wavelength for recording the emission spectra was 561 nm as indicated by the green arrow in (**A**). (**C**) Fluorescence lifetime of an ensemble of SCP complexes immobilized in PVA in semi-logarithmic representation. The full line corresponds to a biexponential fit (see residuals at the bottom) with decay components (amplitudes) of 4.0 ns (85%) and 1.2 ns (15%). The excitation wavelength is 561 nm and the excitation intensity amounts to 525 W/cm^2^. (**D**) Fluorescence lifetimes and spectral peak positions of a single SCP complex. The fluorescence lifetime (scale on the right) was read out every 500 ms and is given by the open dots. The drop out after 4 s presumably results from unresolved blinking. The bin time for recording the spectra was 5.1 s (full line) and the observed spectral peak position of the accumulated spectrum is given by the black dot (scale on the left). The sequence of emission spectra is shown on the right-hand side from top to bottom. For better comparison, the broken vertical line refers to the spectral peak position of the ensemble emission spectrum. (**E**) Two-dimensional representation of the fluorescence lifetime vs. spectral peak position of individual SCP complexes. Due to fluctuations of both lifetime and/or spectral peak position a single complex can contribute more than one data point to the diagram. The number of individual complexes that contribute to the pattern is given in the top left corner. The histograms correspond to the distributions of the spectral peak position (top) and fluorescence lifetime (right). For ease of comparison an ensemble emission spectrum has been overlaid in the top histogram. The statistical parameters of the two histograms (mean ± sdev) are (14,723 ± 109) cm^−1^ for the peak positions, and (3.4 ± 0.8) ns for the lifetimes, respectively.

Samples of unaggregated trimeric SCP complexes dissolved in buffer solution (concentration of 1.45 × 10^–7^ M) have been prepared from fresh samples defrosted on ice under ambient conditions (protocol 1). The corresponding room-temperature absorption spectrum is shown in Fig. 2A by the black line. It features strong peaks in the blue spectral region at 439 nm and 473 nm, and two weaker bands in the red spectral region peaking at 652 nm and 672 nm. The band at 473 nm can be attributed to both the carotenoids and the Chl *b* Soret band, whereas the band at 439 nm is assigned to the absorptions of the Chl *a*/*b* Soret bands^4,32^. The maxima in the red spectral region correspond to the absorptions of the Q_y_ transitions of Chl *b* and Chl *a*, respectively. In order to avoid selective excitation of specific Chl *a*/*b* sites in the SCP complexes we have chosen 561 nm as the excitation wavelength, because this is outside the 650 nm spectral range of the excitonically coupled Chl molecules. For the experiments on single SCP complexes these were immobilised in PVA, and the experiments were carried out at an excitation intensity of 525 W/cm^2^ for obtaining a reasonable signal-to-noise ratio. For better comparison the same excitation intensity has been used also for all experiments on ensembles of SCP complexes immobilised in PVA.

In Fig. 2B the ensemble emission spectra from a solution of SCP complexes (full black line) and from SCP complexes immobilized in a PVA matrix (dashed black line) are compared. Despite the large difference in the excitation intensity used for recording the two spectra, i.e. 30 μW/cm^2^ for the SCP complexes in solution versus 525 W/cm^2^ for those in PVA, both spectra are identical in shape and feature a pronounced peak at 680.0 nm (corresponding to 14,705 cm^−1^; FWHM 383 cm^−1^) accompanied by a low energy wing that extends from 700 to 760 nm. The comparison of the two spectra testifies that the spectral profile of the emission spectrum is neither affected by the immobilization of the SCP complexes in PVA nor by the difference in excitation intensity. The observed transient of an ensemble of SCP complexes embedded in PVA, Fig. 2C, is consistent with a biexponential decay featuring lifetime components of 4 ns, and 1.2 ns with amplitudes of 85%, and 15%, respectively. Using $\tau_{Av} = \frac{{\mathop \sum \nolimits_{i} A_{i} \tau_{i} }}{{\mathop \sum \nolimits_{i} A_{i} }}$, yields 3.6 ns for the amplitude averaged lifetime.

In order to diminish bleaching effects, the experiments on single SCP complexes were conducted under oxygen-free conditions in a vessel that was first flushed with gaseous Argon to remove residual air and then kept under vacuum. In contrast to the experiments reported in^29^ that were conducted using an electrokinetic trap ensuring a well-defined orientation of the pigment-protein complexes, in our experiments the three-dimensional spatial orientation of the SCP complexes is not controlled. Hence, for each individual complex the projection of the absorbing transition-dipole moment on the electric field vector of the excitation light is different leaving the effective excitation intensity unknown. Because of this ambiguity, the emitted intensity is not a reliable parameter for comparing different individual SCP complexes with each other. Instead, we recorded for each individual SCP complex synchronously the fluorescence lifetime and the emission spectrum as a function of time. For doing so, the spectra were read out consecutively every 5.1 s, and the lifetimes were read out consecutively every 500 ms. From these experiments the spectral peak positions of the emission spectra were extracted by fitting a Gaussian to the main peak (see SI). For having the same statistics for the complexes that were studied under different preparation conditions the observation time for each individual complex was restricted to 20 s. If not stated otherwise the accuracy for the lifetimes is limited by the instrument response which amounts to 250 ps, and the accuracy for the spectral peak positions amounts to 0.5 nm (11 cm^−1^).

The data extracted will be displayed in a two-dimensional "lifetimes versus spectral peak positions" diagram, where the horizontal axis corresponds to the spectral peak position and the vertical axis to the fluorescence lifetime, see Fig. 2E. For obtaining these diagrams, periods of both constant peak position and constant lifetime contribute one data point as pointed out in Fig. 2D. The example in Fig. 2D shows on the left-hand side the lifetimes and spectral peak positions as a function of time that have been extracted from an experiment on a single SCP complex. All fluorescence decays were compatible with monoexponentials. (For an explanation that addresses the discrepancy between the monoexponential decays of the single complexes and the non-monoexponential decays observed for the ensembles, see SI). For the first 4 s the fluorescence lifetime amounts to about 4.5 ns before it drops to about 3.4 ns for the remaining observation time. The sharp minimum at about 4 s is attributed to blinking processes that are faster than the 5.1 s bin time for reading out the spectra. For this particular SCP complex the first value is slightly longer than the 3.6 ns that have been found for the average ensemble lifetime. On the right-hand side of Fig. 2D the emission spectra that have been accumulated during the four time intervals are shown. For all spectra the spectral peak positions of 14,684 cm^−1^ are close to the ensemble value and show only little variation as a function of time. For this particular complex we find in total one peak position and two lifetimes, and it therefore contributes two data points to the aforementioned two-dimensional lifetime vs. spectral peak position diagram in Fig. 2E. The two-dimensional diagram with contributions from 31 individual complexes is shown in Fig. 2E, together with the distributions of the spectral peak positions and the fluorescence lifetimes on top of and next to the pattern. The spectral peak positions observed for the individual complexes show only little variation and the corresponding distribution is characterized by a mean of 14,723 cm^−1^ and a standard deviation of 109 cm^−1^. The average peak position is in close agreement with the 14,705 cm^−1^ that was found for the peak of the ensemble spectrum. The distribution of the fluorescence lifetimes is relatively broad, and the statistical parameters amount to 3.4 ns ± 0.8 ns (mean ± sdev), also in good agreement with the average lifetime of 3.6 ns obtained for a large ensemble of SCP complexes.

**Figure 3:**
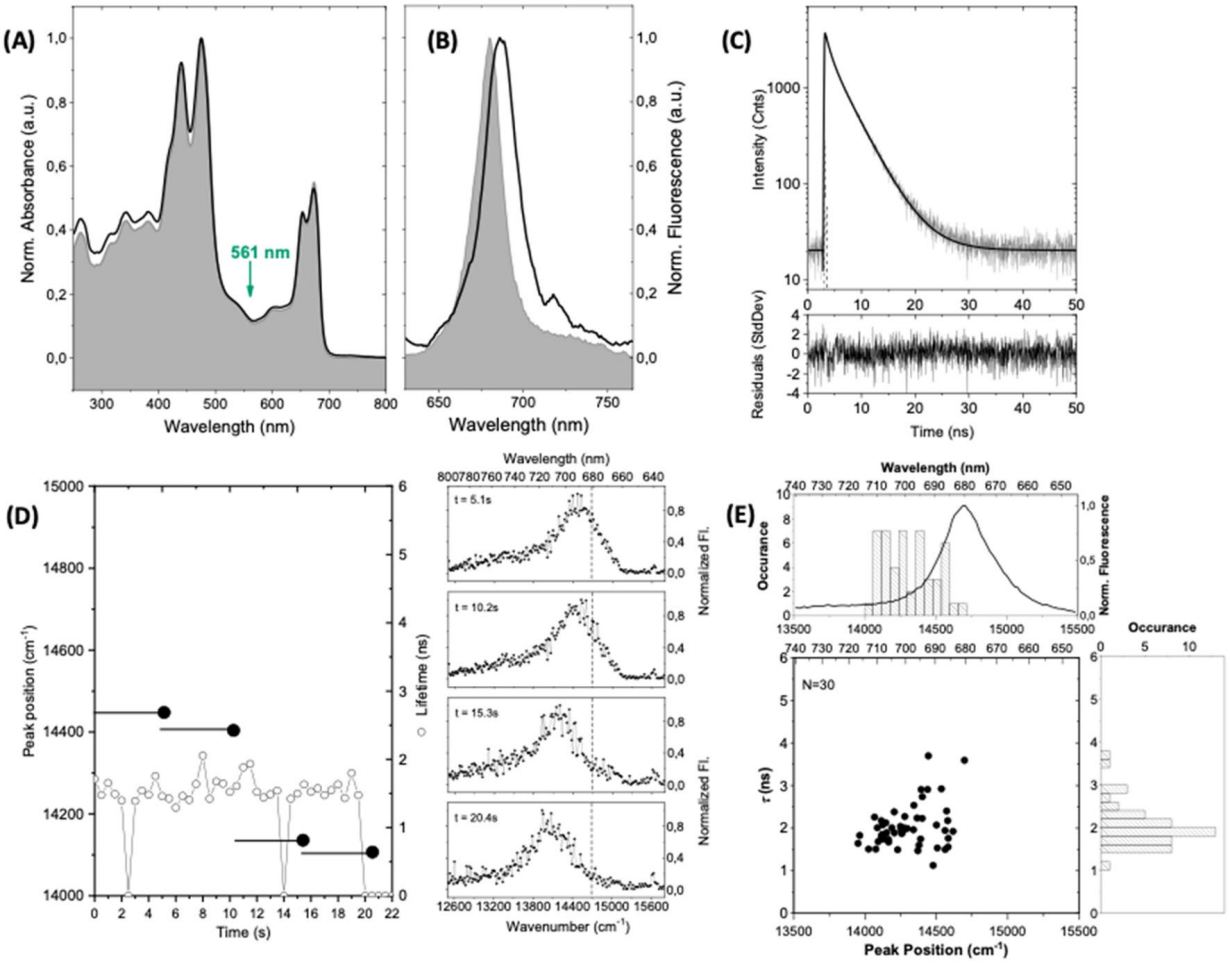
Spectroscopy on ensembles and single SCP complexes that have been stored for 7 months at − 80 °C in the dark. The samples for spectroscopy have been prepared under ambient conditions (protocol 2). (**A**) Room temperature absorption spectrum of an ensemble of SCP that has been stored for 7 months at − 80 °C in the dark dissolved in bulk buffer solution at a concentration of 1.45 × 10^–7^ M (black line). For comparison the corresponding absorption spectrum from the fresh sample (cf. Fig. 2A) has been underlaid as filled spectrum. Both spectra have been peak normalised at 476 nm. (**B**) Emission spectrum from SCP complexes embedded in PVA for an excitation intensity of 525 W/cm^2^. The filled area shows the emission spectrum from a sample of fresh SCP complexes embedded in PVA. Both spectra have been peak normalised for better comparison. The excitation wavelength for recording the emission spectrum was 561 nm as indicated by the green arrow in (**A**). (**C**) Fluorescence lifetime of an ensemble of the 7 months old SCP complexes immobilized in PVA in semi-logarithmic representation. The full line corresponds to a biexponential fit (see residuals at the bottom) with decay components (amplitudes) of 4.3 ns (33%) and 1.2 ns (67%). The excitation wavelength is 561 nm, and the excitation intensity amounts to 525 W/cm^2^. (**D**) Fluorescence lifetimes and spectral peak positions of a single SCP complex. The fluorescence lifetime (scale on the right) was read out every 500 ms and is given by the open dots. The dropouts after 2.5 s and 14 s presumably result from unresolved blinking. The bin time for recording the spectra was 5.1 s (full line) and the observed spectral peak position of the accumulated spectrum is given by the black dot (scale on the left). The sequence of emission spectra is shown on the right-hand side from top to bottom. For better comparison, the broken vertical line refers to the spectral peak position of the ensemble emission spectrum. (**E**) Two-dimensional representation of the fluorescence lifetime vs. spectral peak position of individual SCP complexes. Due to fluctuations of both lifetime and/or spectral peak position a single complex can contribute more than one data point to the diagram. The number of individual complexes that contribute to the pattern is given in the top left corner. The histograms correspond to the distributions of the spectral peak position (top) and fluorescence lifetime (right). For ease of comparison an ensemble emission spectrum from the fresh sample embedded in PVA has been overlaid in the top histogram. The statistical parameters of the two histograms (mean ± sdev) are (14,319 ± 178) cm^−1^ for the peak positions, and (2.0 ± 0.5) ns for the lifetimes, respectively.

Similar experiments have been conducted on SCP complexes from the same batch that has been stored for 7 months at − 80 °C in the dark, and that were prepared under the same conditions as before (protocol 2). The room-temperature absorption spectrum from a solution of these complexes is shown in Fig. 3A by the black line. For reference the absorption spectrum obtained from the fresh sample is underlaid as a filled spectrum. Apart from very small changes below 400 nm the absorption spectra obtained for the different protocols are identical. In Fig. 3B the full line corresponds to the emission spectrum from an ensemble of these SCP complexes embedded in PVA, which is compared with the emission spectrum obtained from the fresh sample that is again underlaid as a filled spectrum. In contrast, to the absorption spectrum the ensemble emission spectrum from the 7 months old sample is red shifted with respect to the initial spectrum and peaks at 686.6 nm (14,564 cm^−1^). Moreover, its width (FWHM) is increased to 440 cm^−1^, and the relative intensity of the low-energy shoulder in the 710 nm to 760 nm spectral range has risen significantly. The decay of the fluorescence from the ensemble is again consistent with a biexponential with lifetimes (amplitudes) of 4.3 ns (33%), and 1.2 ns (67%). While the decay times are about the same as those observed before, their relative weight has shifted towards the short-lived component which is reflected in an averaged lifetime of 2.2 ns. A tentative explanation for this observation is to consider two prevailing conformations of the complexes, each associated with one of the lifetime components. Variations of the population ratio of these conformations will directly impact on the corresponding amplitudes of the two decay components, yet without affecting the time constants.

An example for a single complex is shown in Fig. 3C. The setup of the figure is similar to the setup of Fig. 2C. For this complex the fluorescence lifetime amounts to 1.6 ns during the whole observation time, which is even shorter than the average lifetime of 2.2 ns found for an ensemble under the same conditions. The spectral peak position shows a significant shift from about 14,430 cm^−1^ during the first 10 s to about 14,120 cm^−1^ during the last 20 s, which both are red shifted with respect to the ensemble value. Hence, for this complex we find two peak positions and one lifetime, and this complex contributes two data points to the corresponding two-dimensional lifetime vs. peak position diagram shown in Fig. 3E. For the SCP complexes that have been prepared according to protocol 2 the "peak position versus lifetime" diagram, and the resulting histograms with contributions from 31 individual complexes are shown in Fig. 3E. The distribution of the spectral peak positions does not show a pronounced maximum but is red shifted and clearly broader than the histogram obtained from the fresh complexes. This is also reflected by the statistical parameters of 14,319 cm^−1^ ± 178 cm^−1^. Interestingly, now the histogram for the fluorescence lifetimes is relatively narrow and shifted towards shorter decay times. The statistical parameters for this distribution amount to 2.0 ns ± 0.5 ns, which is in the range of the ensemble average for this preparation protocol.

**Figure 4:**
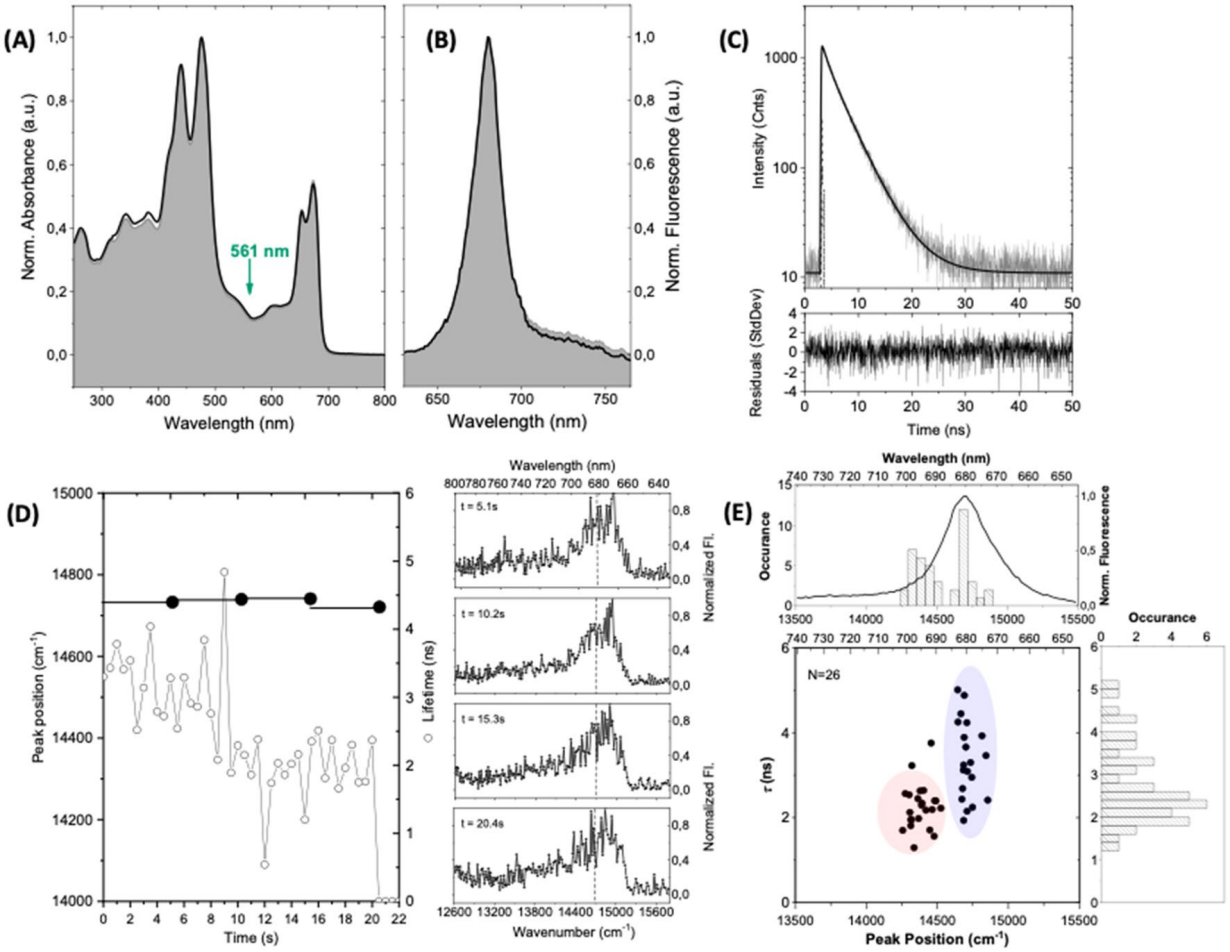
Spectroscopy on ensembles and single SCP complexes that have been stored for 7 months at − 80 °C in the dark. The samples for spectroscopy have been prepared in a cold room at 5 °C (protocol 3). (**A**) Room temperature absorption spectrum of an ensemble of SCP dissolved in bulk buffer solution at a concentration of 1.45 × 10^–7^ M (black line). For comparison the corresponding absorption spectrum from the fresh sample (cf. Fig. 2A) has been underlaid as filled spectrum. Both spectra have been peak normalised at 476 nm. (**B**) Emission spectrum from SCP complexes embedded in PVA for an excitation intensity of 525 W/cm^2^. The filled area shows the emission spectrum from a sample of fresh SCP complexes embedded in PVA. Both spectra have been peak normalised for better comparison. The excitation wavelength for recording the emission spectrum was 561 nm as indicated by the green arrow in (**A**). (**C**) Fluorescence lifetime of an ensemble of SCP complexes immobilized in PVA in semi-logarithmic representation. The full line corresponds to a biexponential fit (see residuals at the bottom) with decay components (amplitudes) of 4.0 ns (79%) and 1.3 ns (21%). The excitation wavelength is 561 nm, and the excitation intensity amounts to 525 W/cm^2^. (**D**) Fluorescence lifetimes and spectral peak positions of a single SCP complex. The fluorescence lifetime (scale on the right) was read out every 500 ms and is given by the open dots. The bin time for recording the spectra was 5.1 s (full line) and the observed spectral peak position of the accumulated spectrum is given by the black dot (scale on the left). The sequence of emission spectra is shown on the right-hand side from top to bottom. For better comparison, the broken vertical line refers to the spectral peak position of the ensemble emission spectrum. (**E**) Two-dimensional representation of the fluorescence lifetime vs. spectral peak position of individual SCP complexes. Due to fluctuations of both lifetime and/or spectral peak position a single complex can contribute more than one data point to the diagram. For the distinction between the data points in the red and blue shaded areas see text. The number of individual complexes that contribute to the pattern is given in the top left corner. The histograms correspond to the distributions of the spectral peak position (top) and fluorescence lifetime (right). For ease of comparison an emission spectrum from a sample of fresh SCP complexes embedded in PVA has been overlaid in the top histogram. The statistical parameters of the two histograms (mean ± sdev) are (14,537 ± 181) cm^−1^ for the peak positions, and (2.8 ± 0.9) ns for the lifetimes, respectively.

Finally, experiments have been conducted on the 7 months old SCP complexes where all preparation steps including spin coating as well as flushing and evacuation of the sample chamber took place in a cold room at 5 °C (protocol 3). The experiments were carried out immediately after the preparation of the sample. The experimental results are summarized in Fig. 4 which has a similar layout as Figs. 2 and 3. The room temperature absorption spectrum from a solution, Fig. 4A, resembles closely the one obtained from the fresh sample, also for the spectral range below 400 nm Fig. 4A. Interestingly, this holds true as well for the emission spectrum that features a peak position at 679.6 nm (14,715 cm^−1^) and a width (FWHM) of 395 cm^−1^ Fig. 4B, and which reproduces the spectral profile observed for the fresh sample. Similarly for the fluorescence decay with lifetime components (amplitudes) of 4.0 ns (79%), and 1.3 ns (21%). The relative weight of the two decay components is nearly restored to the values observed for the fresh SCP complexes. Accordingly, the averaged lifetime amounts to 3.4 ns. The fluorescence lifetime from the single complex shown in Fig. 4D amounts to 3.2 ns for the first 9 s and then drops to 2.0 ns for the remaining duration of the experiment. During this time the spectral peak position does not show significant variations and is centred around 14,735 cm^−1^ corresponding to a slight blue shift with respect to the ensemble value. As before such a complex contributes 2 data points to the respective two-dimensional diagram shown in Fig. 4E.

The statistical parameters for the full dataset with contributions from 26 individual SCP complexes are 14,537 cm^−1^ ± 181 cm^−1^ for the peak positions and 2.8 ns ± 0.9 ns for the fluorescence lifetimes. However, closer inspection of the two-dimensional representation of the data suggests that this diagram represents a superposition of the two patterns observed before for the fresh SCP complexes and for those that have been stored for 7 months in the cold, cf. Figs. 2 and 3. This is corroborated by the separate evaluation of the statistics of the two clusters of data points that are indicated by the blue and red-shaded areas in Fig. 4E. For convenience these will be referred to as the blue and the red cluster in the following. For the data in the blue cluster, the distribution of the peak position is characterised by 14,715 cm^−1^ ± 60 cm^−1^ and that for the lifetimes by 3.4 ns ± 0.9 ns. These numbers are both in very close agreement with the parameters found for the fresh sample. The corresponding parameters for the red cluster amount to 14,382 cm^−1^ ± 77 cm^−1^ for the peak positions and 2.1 ns ± 0.4 ns for the lifetimes. The mean for the lifetimes is close to the value found for SCP complexes that were prepared according to protocol 2, whereas the mean for the spectral peak positions is more shifted to the red. However, the most intriguing observation in the experiments on these SCP complexes was that the classification whether a result felt into the blue or red cluster correlated with the duration of the total experiment. Starting the experiment on a sample immediately after the preparation in the cold room yielded spectral peak positions and fluorescence lifetimes that belonged to the blue cluster, whereas after about 120 min duration of the experiments the observed combination of spectral peak positions and fluorescence lifetimes felt into the red cluster. Jumps of individual complexes between the two clusters were not observed. The experimental results for the three protocols are summarized in Table 1.

**Table 1:** Summary of the spectroscopic results from SCP complexes as a function of the sample preparation conditions. For the ensemble data the spectral peak positions and the widths (FWHM) of the emission spectra are provided. For the single complex data, the means and standard deviations of the respective distributions are given. The excitation intensity at 561 nm was always 525 W/cm^2^.

Ensembles		Protocol 1		Protocol 2		Protocol 3				
Spectral peak position (cm^−1^)		14,705 cm^−1^		14,564 cm^−1^		14,715 cm^−1^				
Spectral peak position (nm)		680.0		686.6		679.6				
FWHM (cm^−1^)		383		440		395				
Lifetimes (ns) (amplitudes)		4.0 (85%) 1.2 (15%)		4.3 (33%) 1.2 (67%)		4.0 (79%) 1.3 (21%)				
Averaged lifetime (ns)		3.6		2.2		3.4				
Single complexes							Protocol 3 blue cluster		Protocol 3 red cluster	
Spectral peak position mean ± sdev (cm^−1^)		14,723 ± 109		14,319 ± 178		14,537 ± 180	14,715 ± 60		14,382 ± 77	
Mean of spectral peak position (nm)		679.2		698.4		687.9	679.6		695.3	
Lifetime mean ± sdev (ns)		3.4 ± 0.8		2.0 ± 0.5		2.8 ± 0.9	3.4 ± 0.9		2.1 ± 0.4	

**Figure 5:**
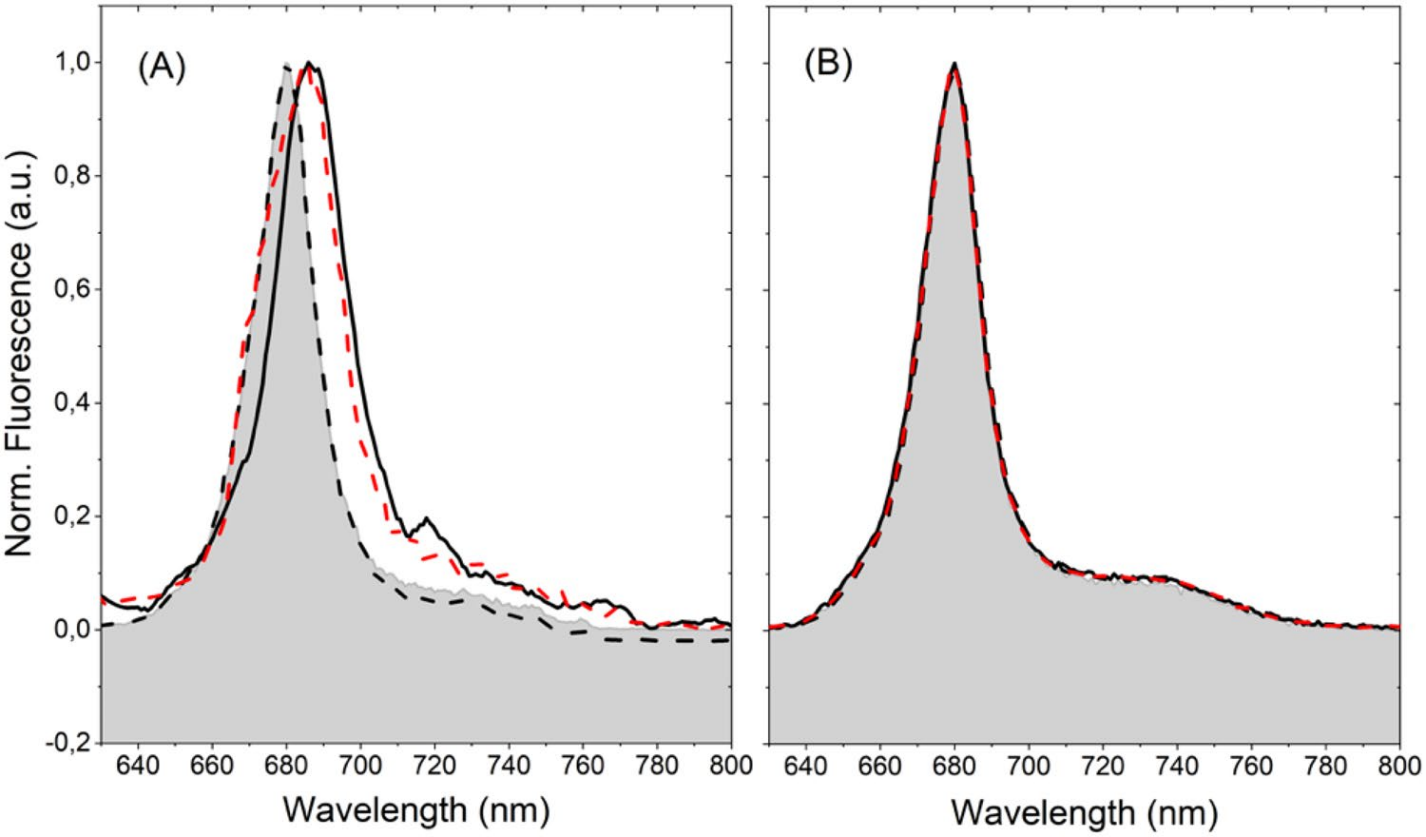
Emission spectra of ensembles of SCP complexes (**A**) embedded in PVA for excitation intensities of 525 W/cm^2^, and (**B**) dissolved in bulk buffer solution at a concentration of 1.45 × 10^–7^ M, and excitation intensities of 30 µW/cm^2^. Key: Dashed black lines—emission spectra for protocol 3; dashed red lines—emission spectra for protocol 3 after a waiting time of 2 h. For ease of comparison, the emission spectra for protocols 1 and 2 are shown by the underlaid filled areas, and the full black lines, respectively.

The experiments on single SCP complexes were carried out using an excitation intensity that exceeds by far the (spectrally integrated) intensity of less than 100 μW/cm^2^ from the sun on a bright day in the natural habitat of the algae. In order to test whether the observed changes of the spectral signatures correlate with the excitation intensity it would be desirable to repeat the experiments using a lower excitation intensity. Due to signal-to-noise restrictions this, however, is only possible for ensembles of SCP complexes. In Fig. 5 the ensemble emission spectra for the various protocols are compared as a function of the excitation intensity. At high excitation intensity, Fig. 5A, the spectrum recorded according to protocol 3 initially reproduces the "protocol 1 like" spectrum (dashed black line), yet changes into the "protocol 2 like" spectrum (red dashed line) after a waiting time (without illumination) of about 2 h. These observations are consistent with the results obtained on single SCP complexes for the different protocols. At lower excitation intensities, Fig. 5B, the emission spectra for all protocols are identical, and moreover reproduce the emission spectrum obtained for protocol 1 at high excitation intensity. This suggests that the formation of the red-shifted quenched state correlates with the excitation intensity.

## Discussion

We have detected the fluorescence lifetimes and the emission spectra from ensembles and single SCP complexes using three different protocols for handling the samples. Taking the data from the fresh sample (protocol 1) as a reference, protocol 2 yields a red shifted ensemble emission spectrum featuring a pronounced low energy shoulder, and a shortening of the average fluorescence lifetime, whereas these parameters are nearly unchanged for applying protocol 3. Since the results obtained from the fresh samples are reproduced for protocol 3, we conclude that the SCP complexes are still intact immediately after taking them out of the low temperature storage. One might argue that the relatively high excitation intensity of 525 W/cm^2^ causes some damage to the SCP complexes. However, we note that at 561 nm this intensity produces about the same number of excited states per unit time as an intensity of 250 W/cm^2^ at 640 nm, which is a common intensity used for spectroscopy of single LHC complexes from green plants^26,27,33,34^. Since such a red-shifted emission has been observed before for LHC II from green plants^34^, and because we do not observe any spectral features that are indicative of free Chlorophyll, namely a fluorescence lifetime of about 5–8 ns^35–37^ and an emission band around 675 nm^38^, we exclude that the emission from the red-shifted quenched state is caused by a deterioration of the samples. Moreover, changes in the PVA matrix causing this shift are excluded as well. Firstly, for all three sample preparation scenarios the PVA was added during the last step of the procedure. Hence, any presumed influence of the PVA on the outcome of the experiments should be identical for all three scenarios. Secondly, in previous work on single light-harvesting complexes from purple bacteria it was shown that the experimental results obtained from complexes that were embedded in PVA and the results from complexes that were reconstituted into a phospholipid bilayer were equivalent^16,39^.

For all three protocols the fluorescence transients recorded from ensembles of SCP complexes are consistent with biexponentials with decay times that are identical within experimental accuracy, namely around 4 ns and 1.2 ns. The decrease of the averaged lifetime from 3.6 ns to 2.2 ns for protocol 2 results solely from the variation of the relative contributions of the two lifetime components to the total decay. In contrast, the experiments on single SCP complexes reveal that all fluorescence decays of single complexes were compatible with monoexponentials. However, for a single complex the decay time may fluctuate as a function of time, and for the micro ensemble of individual complexes studied the decay times feature a distribution. Moreover, for protocol 3 the single SCP complexes undergo a change in the course of time from "protocol 1 like" results during the first two hours after sample preparation to "protocol 2 like" results later.

For LHC II from green plants fluorescence lifetimes between 1.2–1.4 ns and 3.4–3.8 ns with relative contributions between 10–20% for the fast decay time and 80–90% for the slower one, respectively, have been observed before^40^. The fast component is in very good agreement with the fast component found for SCP, whereas the other component for LHC II is slightly faster with respect to the corresponding lifetime component of SCP. The relative weight of the two contributions, however, closely resembles the partition found for fresh samples of SCP, see results for protocol 1. A reduction of the fluorescence lifetime from about 4 ns to 2 ns has been reported for LHC II complexes in thylakoid membranes^41^ which, however, was not accompanied by a red shift of the emission.

**Figure 6:**
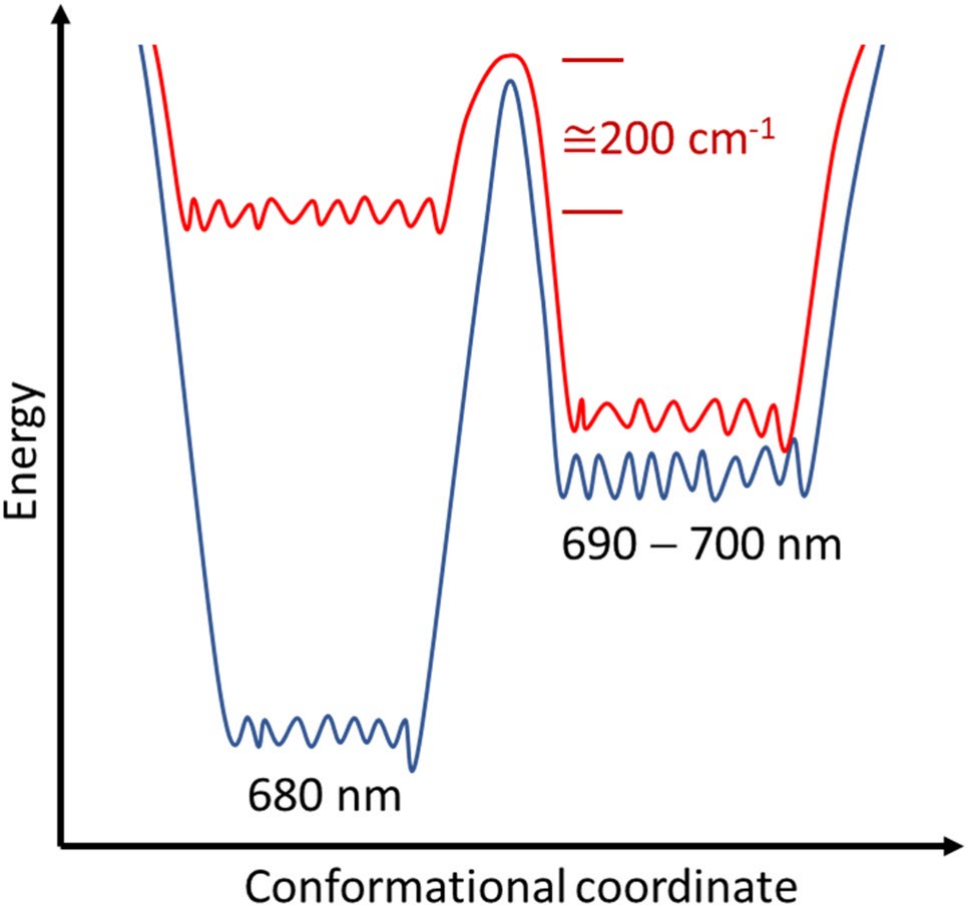
Schematic sketch of the highest tier of the protein energy landscape of the SCP protein structure. For the fresh samples (blue line) the states emitting around 680 nm and around 690–700 nm are separated by a high energy barrier. For the 7 months old samples (red line) the barrier height is lowered such that the available thermal energy at room temperature is sufficient to overcome this threshold.

For single LHC II trimers a reversible switching of the emission between a red-shifted state above 700 nm and a state at 683 nm has been observed before^26,28,34^. Unfortunately, information about the fluorescence lifetimes of these states is not available. The red-shifted emission was associated with a mixed exciton-charge transfer (CT) state that involves the sites Chl *a*603, and Chl *b*609 in close proximity to the lutein 2 molecule, and the appearance/disappearance of this band was ascribed to a conformational change within the protein that affects the mutual interactions of these chromophores^28,34^. Owing to the high degree of homology of the protein structures of SCP and LHC II we hypothesize that in SCP the red-shifted quenched state can be associated as well with structural changes within the protein scaffold that induce the formation of the mixed exciton-charge transfer (CT) state. Given the relatively low fluorescence quantum yield of SCP of 5–10% a large fraction of the average absorbed energy is dissipated by radiationless decay providing a sufficient amount of energy for inducing conformational fluctuations of the protein backbone. This is consistent with the occurrence of the red-emitting state only for higher excitation intensities, see Fig. 5. However, for using high excitation intensities both ensembles and single SCP complexes prepared according to protocol 3 feature a transition from "protocol 1 like" spectra to "protocol 2 like" spectra after a waiting time of about two hours, which presumably reflects the time that is required for warming up the sample chamber to room temperature. From this we infer that for the 7 months old samples the formation of the red-shifted state requires next to a high excitation intensity in addition some thermal activation, and that the corresponding barrier is in the order of the available thermal energy at room temperature, i.e., about 200 cm^−1^. Accordingly, the height of this barrier will be significantly higher than this for the fresh sample (protocol 1), because for this scenario the formation of the red-emitting states was not observed despite using high excitation intensities and handling the samples at room temperature prior to the optical experiments. This hypothesis is sketched in Fig. 6.

The change of the barrier height during storage of the samples could reflect a conformational change that occurs in a part of the protein scaffold such as the formation or the loss of a hydrogen bond, or an isomerization of one of the chromophores or one of the residues in the protein scaffold. As has been shown in^42,43^ for LHC II and a similar light-harvesting structure very little changes in the liganding of the chromophores will have drastic effects for the photophysical properties of the complexes such as spectral position of the emission and/or fluorescence lifetimes. Whether these observations are related to the processes that regulate the photoprotection of the photosynthetic machinery^44^ under strong illumination conditions will need some further attention.

Finally, we note that it is not uncommon to store isolated and purified photosynthetic antennae proteins for some time span at − 80 °C in the dark, and to verify the integrity of the samples by taking UV/VIS spectra. This is usually carried out using low excitation intensities in the order of some 10–100 µW/cm^2^. However, the results presented here for the light-harvesting complexes of the marine alga *Codium fragile* show that this might be problematic and that (at least for this particular light-harvesting complex) ensemble UV/VIS spectroscopy might not be sufficient to verify the intactness of the samples.

## Materials and methods

### Cultivation of algae

*Codium fragile* (KU-065, KU-MACC, Kobe, Japan) was cultivated in floating form in Provasoli’s Enriched Sea water (PES) medium as described in^42^ using artificial sea water (Marine Art SF-1, Tomita Pharmaceutical Co., Ltd, Tokushima, Japan) at 21 °C, and illuminated with a white light LED (AS-010, Fujikura, Osaka, Japan) at 0.5 mW/cm^2^ with 12 h/12 h light cycles.

### Protein preparation/purification

Thylakoid membranes were prepared from lyophilized algae according to^45^. SCP was purified from the thylakoid membranes as detailed in^4^ with slight modifications: After solubilization, the samples were loaded onto the layer of sucrose density gradient as described in^46^. The major band was collected and purified by anion exchange chromatography, see^4^. This step was repeated, and the material was further purified using another sucrose density gradient followed by gel filtration (Sephacryl S-200HR, Merck KGaA, Darmstadt, Germany) with 20 mM Tris–HCl (pH 8.2) containing 0.03% n-dodecyl β-D-maltoside (β-DDM). Purified SCP was flash frozen with liquid nitrogen, stored at − 80 °C, and transported at liquid nitrogen temperature using Dry Shipper (CX100, Taylor-Wharton, Baytown, Tx, USA).

### Sample handling

The SCP samples with an optical density of 0.2 at 670 nm were defrosted on an ice bucket and diluted in 25 mM Tris–HCl buffer pH 8.2 containing 0.03% n-dodecyl β-D-maltoside (β-DDM) to give a 1.45 × 10^–7^ M solution of SCP complexes. For removing oxygen, the buffer was degassed by ultrasonication for 30 min. The solution was immediately divided into aliquots of 10 μL, and either used immediately, or snap-frozen in liquid nitrogen and stored at − 80 °C for later use. All handlings took place in the dark while the samples were kept cold in an ice bucket.

*Protocol 1* Protocol 1 refers to fresh samples. To prepare the sample, the aliquoted SCP solution (1.45 × 10^–7^ M) was defrosted on an ice bucket. For the experiments on ensembles of SCP complexes embedded in a thin film of polyvinyl alcohol (PVA) about 1 µL from the aliquots was further diluted with the buffer to a volume of 200 µL and 2% of PVA (w/v; mw 124.000–186.000) were added. For the experiments on the single SCP complexes the sample was further diluted with the buffer in three steps to 0.07 pM, and in the last dilution step 2% (w/v) PVA was added. Subsequently a small droplet (25 μL) of the solution was spin-coated in the dark onto a glass substrate (for 10 s at 500 rpm and 60 s at 2000 rpm) forming a thin polymer film of about 100 nm thickness with embedded SCP complexes. Prior to the spin coating the glass substrate was three times carefully cleaned with acetone and dried with a strong flow of nitrogen gas. The substrate served as window of a vacuum chamber with the sample positioned on the inside which allows to work under oxygen-free conditions. Immediately after mounting the window the vacuum chamber was flushed 3 times with gaseous Argon to remove residual air and then evacuated to 10^–3^ mbar.

*Protocol 2* Protocol 2 refers to SCP samples that have been stored at − 80 °C 7–8 months, and that were prepared for the spectroscopic experiments following the same procedures as detailed above for protocol 1.

*Protocol 3* Protocol 3 refers to SCP samples that have been stored at − 80 °C 7–8 months, but where the whole preparation of the samples for the spectroscopic experiments including spin coating, and mounting of the samples was carried out in a cold room (5 °C) in the dark. After mounting the sample chamber was evacuated, moved to the optical setup, flushed with gaseous Argon and then evacuated again.

### Optical experiments

For recording the UV/VIS spectra the dissolved SCP complexes at a concentration of 1.45 × 10^–7^ M were filled into quartz-glass cuvettes (Hellma QS) that was mounted in commercial spectrometers (absorption: Perkin Elmer Lambda75, emission: Varian Cary Eclipse spectrometer). The emission spectrum was recorded for an excitation wavelength of 561 nm and an intensity that corresponded to 30 µW/cm^2^. The single-complex experiments were conducted on a commercial single-molecule optical microscope (MicroTime 200, PicoQuant). The SCP complexes were excited with a laser diode at 561 nm (LHD-D-TA-560B, PicoQuant) operated in pulsed mode with a repetition rate of 20 MHz and an intensity of 525 W/cm^2^. The output from the laser was coupled into an optical fibre and entered an inverted confocal optical microscope where it was focused with a water immersion objective (60 × UPlanS APO UIS2, NA = 1.2, Olympus) onto the sample. In order to find a single complex, the focal spot on the sample could be scanned over a range of 250 µm × 250 µm using a galvo scanner (FLIMbee unit, PoicoQuant). The emission from the sample passed a dichroic mirror (ZT488/561 kpc, AHF/Croma) and suitable optical filters (LP H 560 LPXR, AHF) and was then split in a beamsplitter cube in a ratio of 50/50. One part of the signal was directed towards a spectrometer (Shamrock SR-163, grating 600 lines/mm blazed at 500 nm) via another fibre, and the emission spectrum was detected using an electron-multiplying charge-coupled device (EMCCD Newton 970, Andor). The spectral resolution was 0.5 nm corresponding to 11 cm^−1^ at 680 nm. The other half of the signal was focused onto a single-photon counting avalanche diode (SPCM-AQRH-14-TR, Excelitas) and used for time tagged time-resolved (TTTR) fluorescence data collection employing time correlated single photon counting (TCSPC TimeHarp 260 PICO Dual, PicoQuant). The transients were measured with a temporal resolution of 250 ps. In order to avoid pile-up effects in the photon counting statistics when measuring ensembles, we ensured that the detection count rate was less than 1% of the laser repetition rate by placing an OD 3 in front of the detector. For obtaining the fluorescence lifetimes the measured transients were deconvoluted with the instrument response function using commercial software (SymPhoTime 64, Picoquant). All experiments have been conducted at room temperature.

### Fluorescence quantum yield measurement

The fluorescence quantum yields were measured and determined according to the procedure described in^47^. The measurements on SCP were carried out at room temperature using a laser diode (Thorlabs, HL6358MG) operating at 639 nm as excitation light source. For detection a CCD-camera (ANDOR IDUS 420) that was connected to a spectrograph (MS125), which in turn was fibre-coupled to an integrating sphere was used.

## Supplementary Information

Supplementary Information.
