# Supplementary material for: Preprocess dependence of optical properties of ensembles and single siphonaxanthin-containing major antenna from the marine green alga Codium fragile
Source: Sci Rep. 2022 May 19;12:8461. doi: 10.1038/s41598-022-11572-3 (PMC9120457; doi:10.1038/s41598-022-11572-3)
Supplement: Supplementary file 1 — Supplementary Information. [file 41598_2022_11572_MOESM1_ESM.docx]

Supplementary Information for:

**Preprocess dependence of optical properties of ensembles and single siphonaxanthin-containing major antenna from the marine green alga *Codium fragile***

Tatas Hardo Panintingjati Brotosudarmo^1,2^, Bernd Wittmann^1^, Soichiro Seki^3^,

Ritsuko Fujii^3,4^, and Jürgen Köhler^1,5,6,*^

^1^Spectroscopy of soft Matter, University of Bayreuth, 95440 Bayreuth, Germany; tatas.brotosudarmo@uni-bayreuth.de (THPB), bernd.wittmann@uni-bayreuth.de (BW), juergen.koehler@uni-bayreuth.de (JK)

^2^ Department of Food Technology, Universitas Ciputra, Citraland CBD Boulevard, Surabaya 60219, Indonesia; tatas.brotosudarmo@ciputra.ac.id (THPB)

^3^Graduate School of Science, Osaka City University, 3-3-138 Sugimoto, Sumiyoshi-ku, Osaka 558-8585, Japan; sekiso@sci.osaka-cu.ac.jp (SS); ritsuko@osaka-cu.ac.jp (RF)

^4^Research Center for Artificial Photosynthesis, Osaka Metropolitan University, 3-3-138 Sugimoto, Sumiyoshi-ku, Osaka 558-8585, Japan; ritsuko@osaka-cu.ac.jp (RF)

^5^Bavarian Polymer Institute, University of Bayreuth, 95440 Bayreuth, Germany, juergen.koehler@uni-bayreuth.de (JK)

^6^Bayreuther Institut für Makromolekülforschung (BIMF), 95440 Bayreuth, Germany; juergen.koehler@uni-bayreuth.de (JK)

*Correspondence: juergen.koehler@uni-bayreuth.de

Contents

1. Examples of Gaussian fits of the emission spectra
2. Monoexponential decays for single complexes vs. non-monoexponential decays for ensembles

**1. Examples of Gaussian fits of the emission spectra**

The spectral peak positions of the emission spectra were determined by fitting the main peak with a Gaussian profile. Examples are shown in fig.S1.


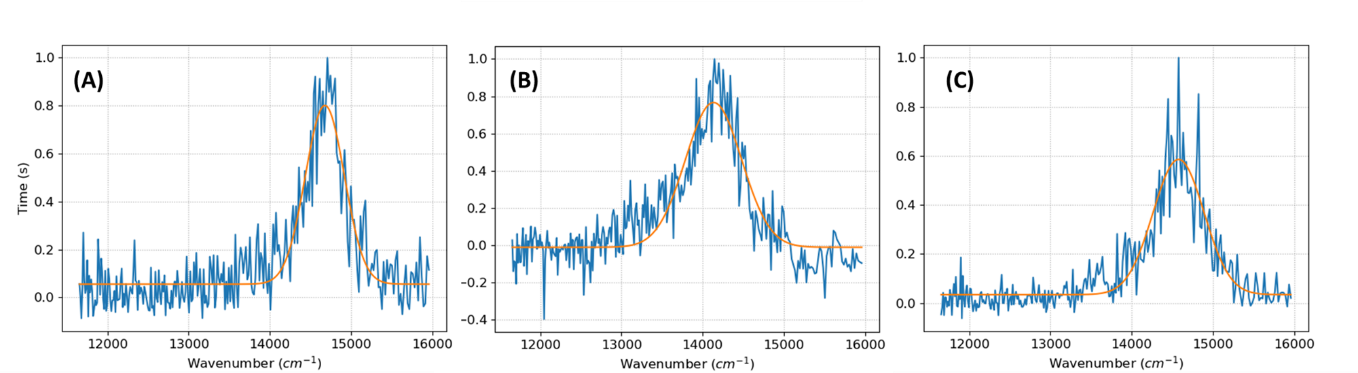


Figure S1: Examples of a Gaussian fits of the emission spectra of single SCP complexes for A) protocol 1, B) protocol 2 C) protocol 3. The excitation wavelength was always 561 nm, and the corresponding intensity 525 W/cm^2^.

**2. Monoexponential decays for single complexes vs. non-monoexponential decays for ensembles**

All fluorescence transients observed from single SCP complexes feature a monoexponential decay whereas those from the ensembles are not monoexponential. This discrepancy is not uncommon when comparing lifetimes from single objects with those from ensembles. Consider an ensemble of objects where the individual objects feature monoexponential decays with slightly different lifetimes with respect to each other. In other words, the lifetimes from the set of individual objects are subjected to a distribution as for example shown in parts E) of figs.2-4 in the manuscript. Then the fluorescence decay of the ensemble reflects the superposition of all individual contributions and will deviate from a monoexponential decay. The best would be to fit the ensemble decay with the appropriate distribution of lifetimes. Usually, this distribution is unknown and the simplest approximation to a non-monoexponential decay is a bi-exponential decay. As long as the values for the two lifetimes are not too close to each other this is a good, reproducible representation of the overall lifetimes.
